# Supplementary figures and images for: Gene expression associated with white syndromes in a reef building coral, Acropora hyacinthus
Source: BMC Genomics. 2015 May 9;16(1):371. doi: 10.1186/s12864-015-1540-2 (PMC4425862; doi:10.1186/s12864-015-1540-2)

DEGs FDR < 10%

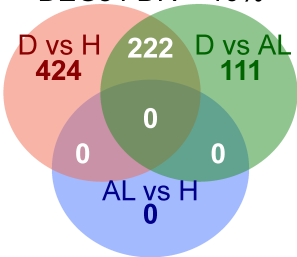

Supplement: Additional file 2: — Venn diagram of DEGs passing the FDR threshold of 10% for each of the contrasts. The largest number of DEGs resulted from the contrast of diseased (D) tissue to healthy (H) tissues, followed by the contrast of diseased to tissues ahead of the lesion (AL). No isogroups passed the FDR cutoff for the AL-healthy contrast. [file 12864_2015_1540_MOESM2_ESM.pdf]

## MF Disease vs AL

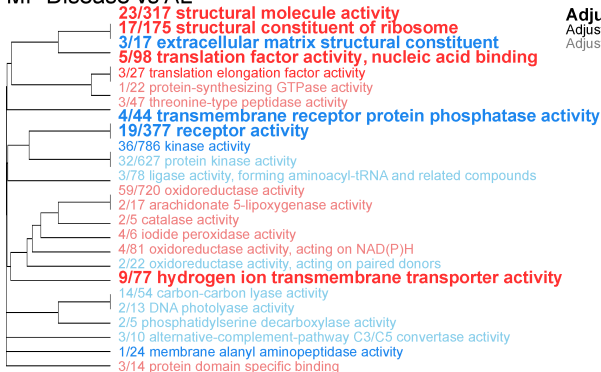

## BP Disease vs AL

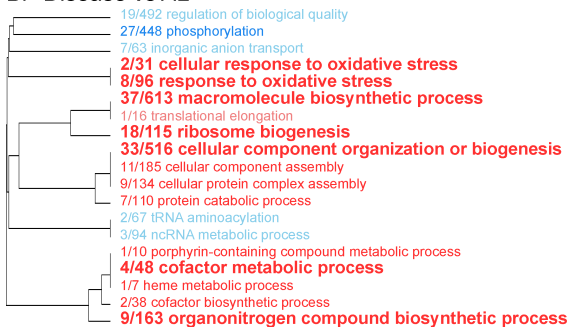

## CC Disease vs AL

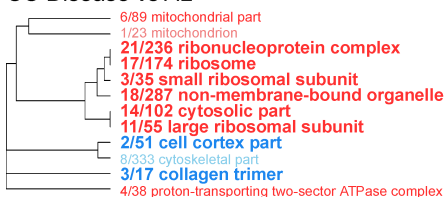

Supplement: Additional file 3: — Gene ontology categories enriched by genes up-regulated (red) or down-regulated (blue) in diseased compared to AL samples, summarized by molecular function (MF), biological process (BP), and cellular component (CC). The size of the font indicates the significance of the term as indicated by the inset key. The fraction preceding the GO term indicates the number of genes annotated with the term that pass an unadjusted p-value threshold of 0.05. The trees indicate sharing of genes among GO categories (the categories with no branch length between them are subsets of each other). [file 12864_2015_1540_MOESM3_ESM.pdf]

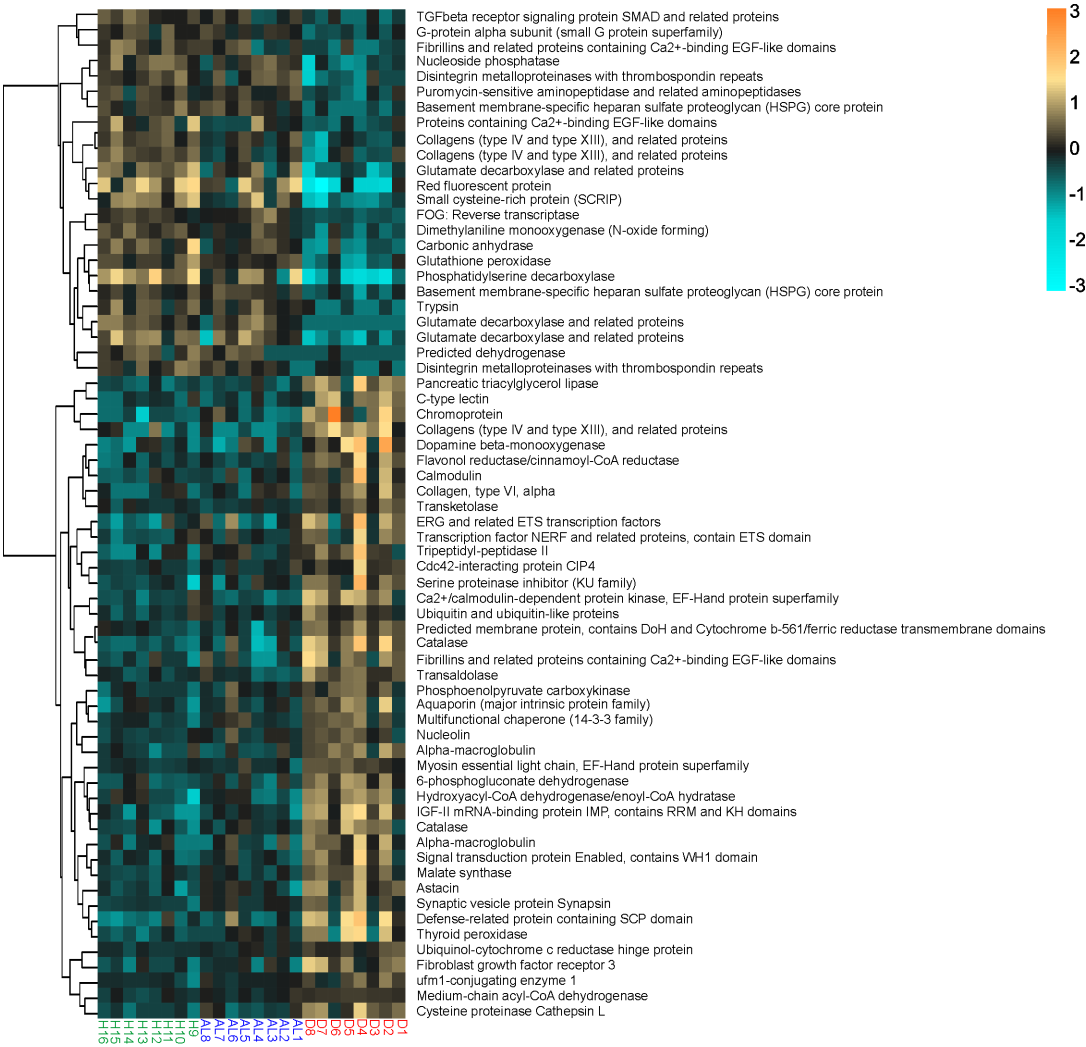

Supplement: Additional file 4: — Gene expression heatmaps for annotated DEGs (adjusted p-value < 0.01) for the disease-healthy contrast. Rows are genes, columns are samples ordered as in the bottom panel: ahead-of-lesion (AL), healthy (H), and diseased (D). The color scale is in log2 (fold change relative to the gene’s mean). The trees are a hierarchical clustering of genes based on Pearson’s correlation of their expression across samples. [file 12864_2015_1540_MOESM4_ESM.pdf]

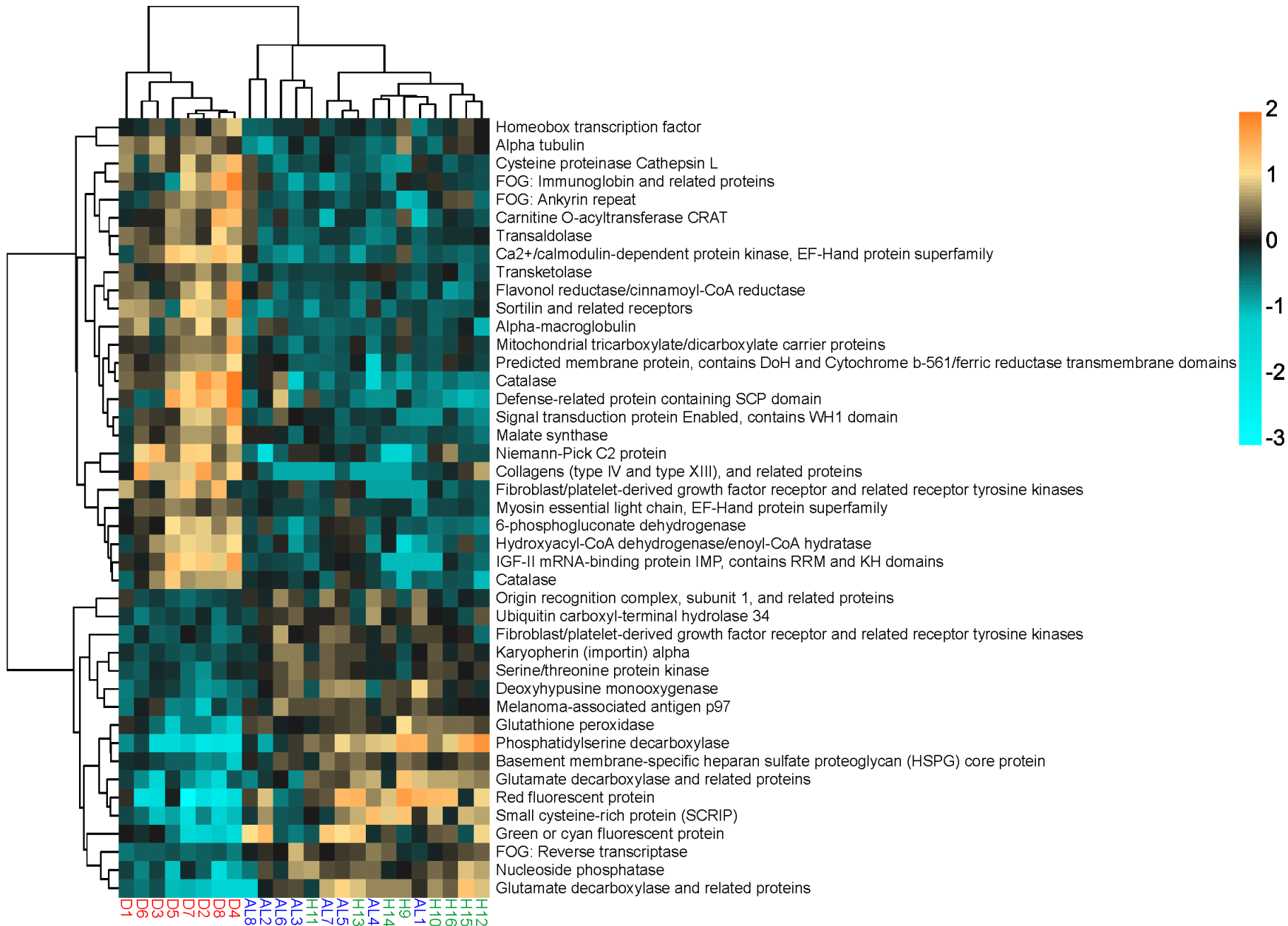

Supplement: Additional file 5: — Gene expression heatmaps for annotated DEGs (unadjusted p-value < 0.01) for the disease-AL contrast. Rows are genes, columns are samples ordered as in the bottom panel: ahead-of-lesion (AL), healthy (H), and diseased (D). The color scale is in log2 (fold change relative to the gene’s mean). The trees are hierarchical clustering of genes based on Pearson’s correlation of their expression across samples. [file 12864_2015_1540_MOESM5_ESM.pdf]

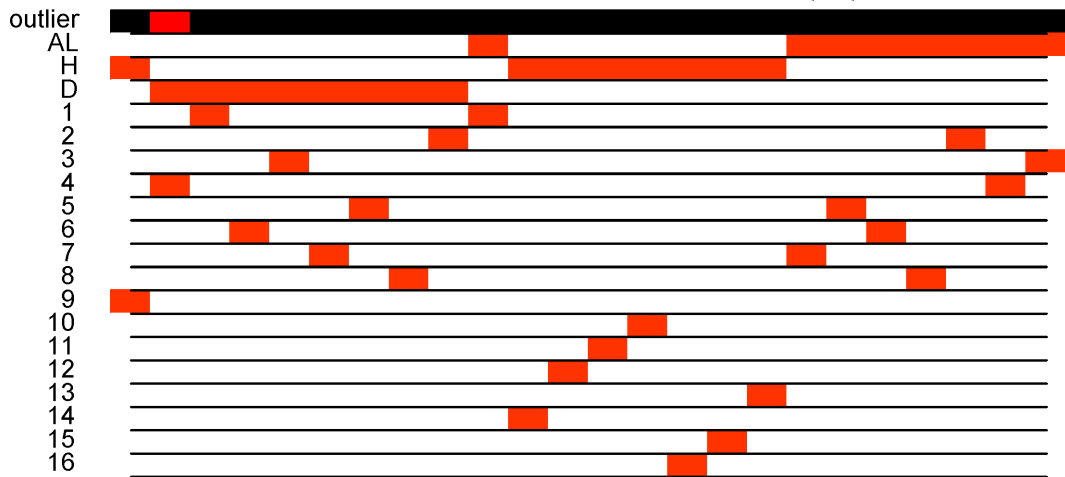

Supplement: Additional file 9: — Sample dendrogram and outlier heatmap for WGCNA. Sample clustering allows the visualization of how traits (health states and individual genotypes in this case) relate to samples. The dendrogram does not present any obvious outliers, but individual “D4” is called as an outlier based on a standardized connectivity test. [file 12864_2015_1540_MOESM9_ESM.pdf]

# Module-trait relationships

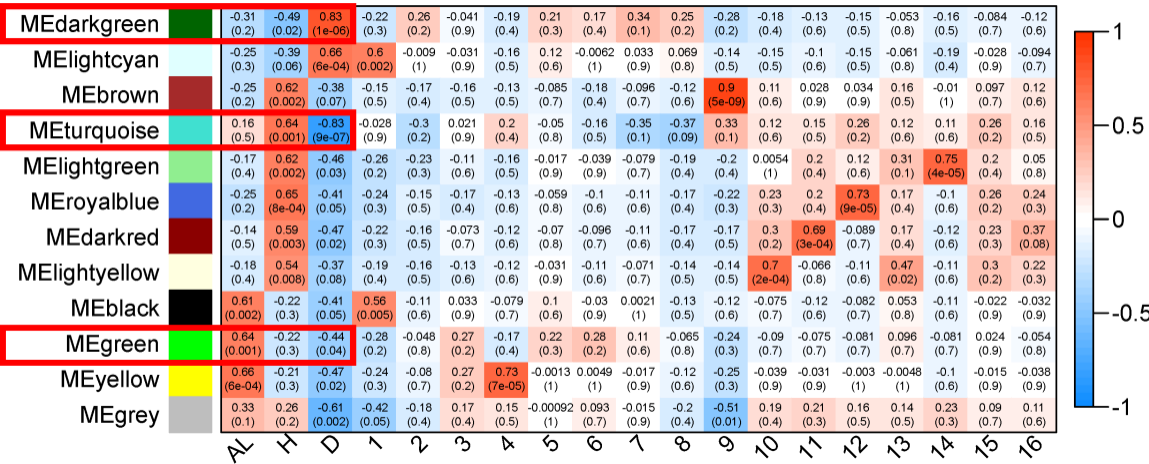

Supplement: Additional file 10: — Heatmap of module-trait correlations. The strength of the correlations between traits (health states or individual corals) and gene coexpression modules are indicated by the intensity of color. The numbers in the cells give Pearson’s correlation between the module eigengene and the trait and the p-value according to the correlation test. Red boxes mark the three modules that are highly and specifically correlated to each of the health states. [file 12864_2015_1540_MOESM10_ESM.pdf]

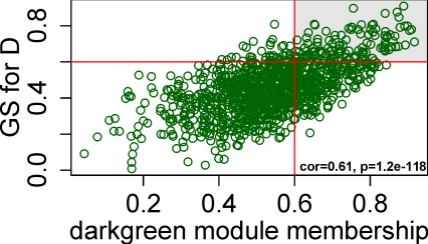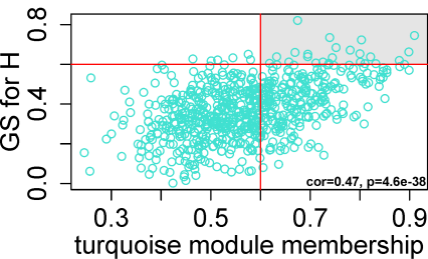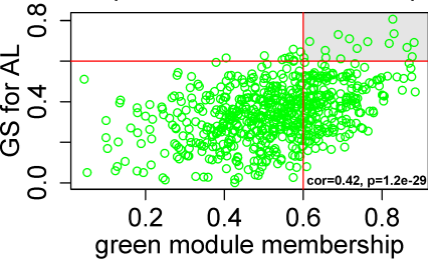

Supplement: Additional file 11: — Dependency between individual genes’ module membership (correlation with module’s eigengene) and significance for the disease state (correlation of the gene with the disease state). The grey region encompasses genes with both module membership and gene significance scores higher than 0.6. Pearson correlation values and the p-value of the correlation test are indicated in the lower-right hand of each scatterplot. [file 12864_2015_1540_MOESM11_ESM.pdf]

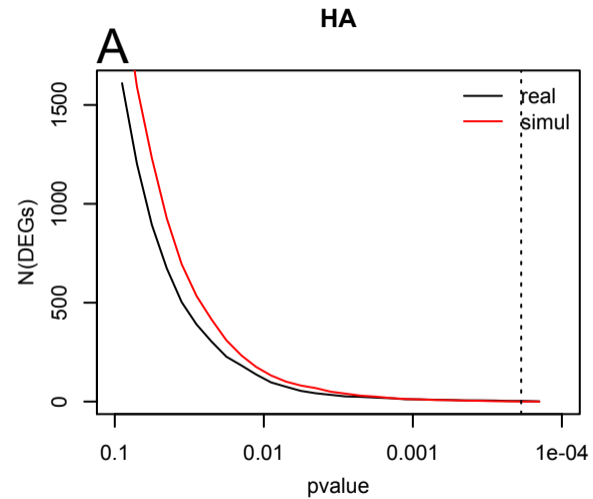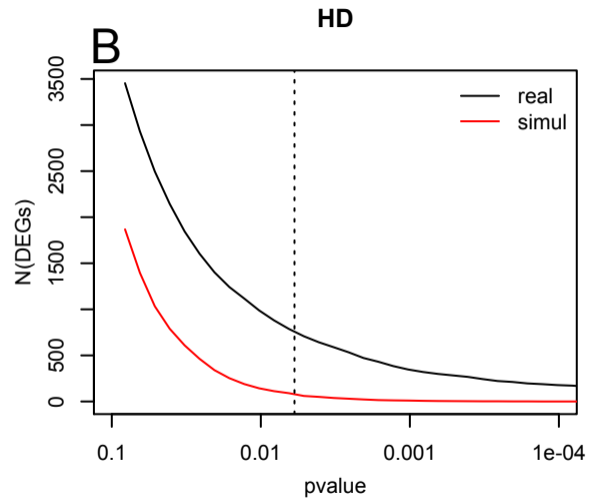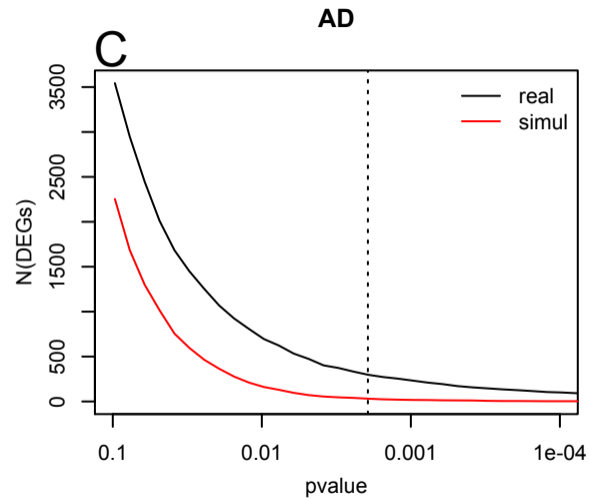

Supplement: Additional file 13: — Calculating empirical false discovery rates using simulated data. The x-axis is the Wald test p-value and the y-axis is the number of differentially expressed genes (DEGs) passing this p-value cutoff. The black line corresponds to DEGs discovered in the real dataset and red line corresponds to DEGs discovered in the simulated dataset (false positives). The vertical dashed line indicates the empirical 10% false discovery rate (FDR) cutoff. (A) Healthy-AL comparison. (B) Healthy-Disease. (C) AL-Disease comparison. [file 12864_2015_1540_MOESM13_ESM.pdf]

# Module-Trait Relationships

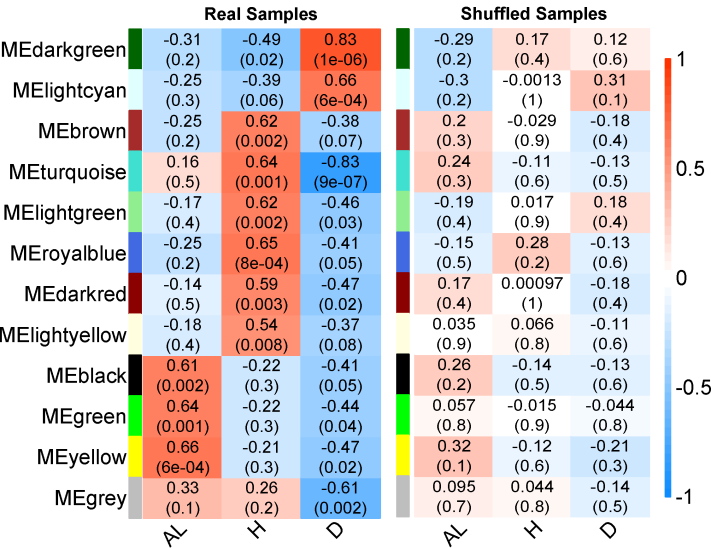

Supplement: Additional file 14: — Shuffling sample-condition assignments dissolves module-trait relationships in WGCNA. On both panels, rows are module eigengenes (MEs) and the columns are traits (AL – ahead of lesion, H – healthy, and D –diseased). The color scale reflects the correlation of the module’s eigengene with the trait. The numbers in the cells are the Pearson r and the p-value of the correlation test (in parentheses). Strong correlations between modules of gene co-expression and health states were identified when the samples were assigned to correct conditions (left). These correlations disappeared when condition designations were shuffled among samples (right), providing evidence of a true biological relationship between the identified gene coexpression modules and health states. [file 12864_2015_1540_MOESM14_ESM.pdf]
