# Supplementary material for: Gene expression associated with white syndromes in a reef building coral, Acropora hyacinthus
Source: BMC Genomics. 2015 May 9;16(1):371. doi: 10.1186/s12864-015-1540-2 (PMC4425862; doi:10.1186/s12864-015-1540-2)
Supplement: Additional file 12: — Simulation quality plots compare real and simulated DESeq2 datasets. (A) Size factors are nearly identical between real (dds) and simulated (sims) data sets. (B-C) Dispersion estimates of the real data set strongly agree with simulated dispersion estimates. (D) Dispersions are nearly identical between real (dds) and simulated (sims) data sets. (E-F) MA plots of log-fold change by mean expression value of real and simulated datasets are nearly identical. [file 12864_2015_1540_MOESM12_ESM.pdf]

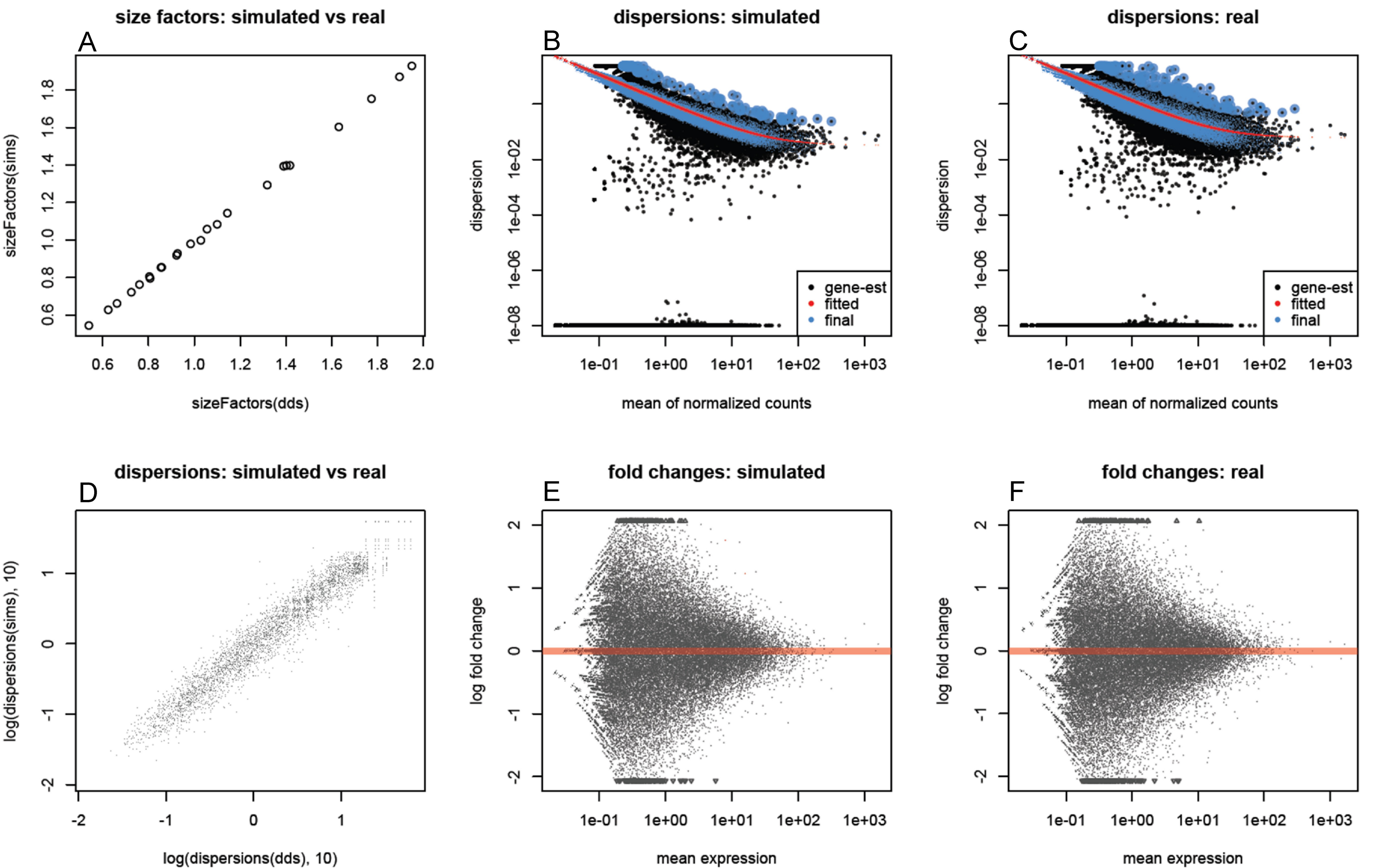

size factors: simulated vs real
A
sizeFactors(sims)
sizeFactors(dds)
dispersions: simulated
B
dispersion
mean of normalized counts
gene-est
fitted
final
dispersions: real
C
dispersion
mean of normalized counts
gene-est
fitted
final
dispersions: simulated vs real
D
log(dispersions(sims), 10)
log(dispersions(dds), 10)
fold changes: simulated
E
log fold change
mean expression
fold changes: real
F
log fold change
mean expression
